# Suppression of stacking fault expansion in a 4H-SiC epitaxial layer by proton irradiation

**DOI:** 10.1038/s41598-022-17060-y

**Published:** 2022-08-15

**Authors:** Shunta Harada, Toshiki Mii, Hitoshi Sakane, Masashi Kato

**Affiliations:** 1 Center for Integrated Research of Future Electronics (CIRFE), Institute of Materials and Systems for Sustainability (IMaSS), Nagoya University, Furo-cho, Chikusa-ku, Nagoya, 464-8601 Japan; 2 Department of Materials Process Engineering, Nagoya University, Furo-cho, Chikusa-ku, Nagoya, 464-8603 Japan; 3 Department of Electrical and Mechanical Engineering, Nagoya Institute of Technology, Nagoya, 466-8555 Japan; 4 SHI-ATEX Co., Ltd., 1501, Imazaike, Saijo-shi, Ehime 799-1393 Japan

**Keywords:** Materials science, Materials for devices, Electronic devices

## Abstract

SiC bipolar degradation, which is caused by stacking fault expansion from basal plane dislocations in a SiC epitaxial layer or near the interface between the epitaxial layer and the substrate, is one of the critical problems inhibiting widespread usage of high-voltage SiC bipolar devices. In the present study, we investigated the stacking fault expansion behavior under UV illumination in a 4H-SiC epitaxial layer subjected to proton irradiation. X-ray topography observations revealed that proton irradiation suppressed stacking fault expansion. Excess carrier lifetime measurements showed that stacking fault expansion was suppressed in 4H-SiC epitaxial layers with proton irradiation at a fluence of 1 × 10^11^ cm^−2^ without evident reduction of the excess carrier lifetime. Furthermore, stacking fault expansion was also suppressed even after high-temperature annealing to recover the excess carrier lifetime. These results implied that passivation of dislocation cores by protons hinders recombination-enhanced dislocation glide motion under UV illumination.

## Introduction

Hexagonal silicon carbide (SiC) with the 4H polytype is a promising semiconductor material for high-power and high-temperature devices^1–3^. Owing to recent progress in SiC device technology, 1-kV-class SiC Schottky barrier diodes (SBDs) and metal–oxide–semiconductor field effect transistors (MOSFETs) have already been commercialized and used in various kinds of electronic systems such as power supplies for servers and workstations, solar inverters, uninterruptible power supplies, industrial motor drives, air-conditioners, fast chargers, elevators, electric vehicles, and railcars^4,5^. On the other hand, SiC bipolar devices such as p-i-n diodes, insulated gate bipolar transistors (IGBTs) and thyristors encounter problems with device degradation, in which the forward voltage is increased due to expansion of single Shockley-type stacking faults (1SSFs) in the epitaxial layer under forward-bias conditions^6–12^. The expansion of 1SSFs originates from extended dislocations having Burgers vector of 1/3<11-20> on the (0001) basal plane, which are called basal plane dislocations (BPDs). 1SSFs between two partial dislocations are expanded during bipolar operation by gliding of partial dislocations, driven by the “negative” stacking fault energy due to the lowering of the electronic energy by carrier trapping at the stacking fault^13–18^. Similarly, double Shockley-type stacking faults (2SSFs) in heavily nitrogen-doped 4H-SiC undergo expansion during high-temperature annealing^19–24^. The anomalous behavior of stacking faults in 4H-SiC is considered to be due to the relatively low stacking fault energy, which was estimated to be 14.7 mJ m^−2^ for 1SSFs^25^, and a large energy gain due to the energy level of localized state in the stacking faults, which is estimated to be 0.22 and 0.59 eV below the conduction band edge of 4H-SiC for 1SSFs and 2SSFs, respectively^20,21^.

Since 1SSF expansion was reported to originate from BPDs in the epitaxial layer, great efforts have been made to reduce the BPD density in epitaxial layers^26–29^. Thanks to dislocation conversion from BPDs to threading edge dislocations (TEDs) propagating in the [0001] direction during the epitaxial growth process, the typical BPD density in commercial SiC epitaxial wafers is almost zero (less than 1 cm^−2^)^3,5,30^. However, 1SSF expansion underneath BPD-TED conversion points under high current stress has been reported^31–33^. To suppress 1SSF expansion, proper design of the buffer layer, which is the first thin layer grown on a substrate, is important. Tawara et al. have clearly demonstrated the relationship between the injected carrier concentration and 1SSF expansion, and showed that a recombination-enhancing buffer layer in p-i-n diodes suppresses 1SSF expansion^33,34^. However, a thick buffer layer is necessary to suppress 1SSF expansion under a high current density, resulting in an increased process cost. Therefore, it is desirable to develop other strategies to suppress stacking fault expansion. Another important aspect of the spontaneous expansion of 1SSFs is a decrease in the critical resolved shear stress (CRSS) for gliding of partial dislocations, called “recombination-enhanced dislocation glide” in 4H-SiC, as reported by many researchers^9,13,35^. Thanks to the drastic decrease in the CRSS, 1SSFs were reported to expand even below room temperature^35^. Considering that the CRSS for basal slip in SiC was estimated to be as large as 5–10 GPa at room temperature without recombination-enhanced dislocation glide^36,37^, the anomalous decrease in the CRSS for partial dislocations is expected to take place under a high current density.

Proton irradiation is widely used in semiconductor processes for the purpose of doping and control of lifetimes, including SiC device processes^38–41^. Proton irradiation results in the formation of radiation-induced defects as well as hydrogen doping. It was reported that the defects created by proton irradiation introduced *Z*_1/2_ deep levels and reduced the carrier lifetime^42–44^. In the present study, we investigated stacking fault expansion in SiC epitaxial layers subjected to proton irradiation. To investigate the stacking fault behavior, we used optical excitation of excess carriers by ultraviolet (UV) illumination to stimulate stacking fault expansion^9,45,46^.

## Experimental procedure

**Figure 1:**
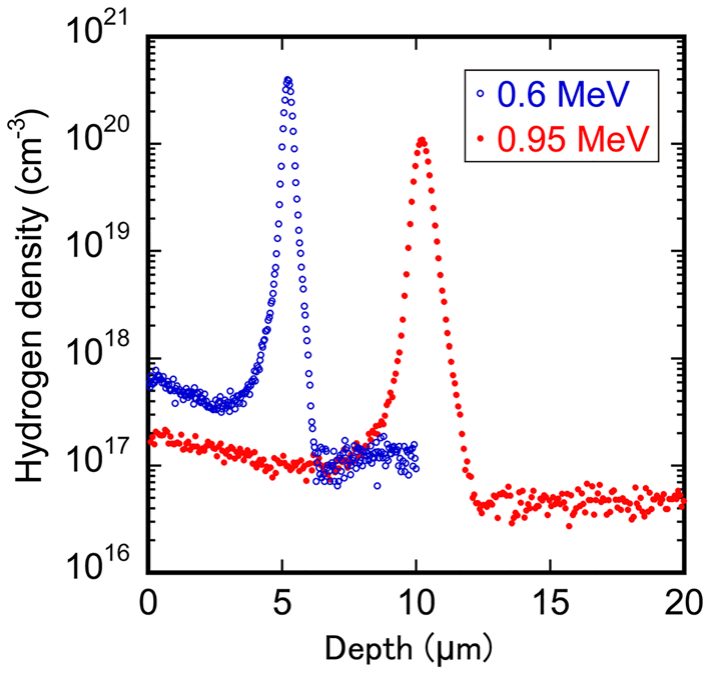
Depth profile of hydrogen density for specimens subjected to proton fluence of 1 × 10^16^ cm^−2^ measured by SIMS.

An N-type 4H-SiC epitaxial layer with a thickness of 10 μm, a nitrogen concentration of 1.0 × 10^16^ cm^−3^ and an off-cut angle of 4° from the (0001) basal plane was grown by chemical vapor deposition (CVD) on a SiC wafer (SiCrystal GmbH), which was then cut by laser scribing to give specimens 5–10 mm in length. The specimens were irradiated at room temperature with 0.6-MeV and 0.95-MeV protons at fluences ranging from 1 × 10^11^ to 1 × 10^16^ cm^−2^. The hydrogen distribution in a specimen irradiated with a fluence of 1 × 10^16^ cm^−2^ was investigated by secondary ion mass spectrometry (SIMS), and the results are shown in Fig. 1. The maximum hydrogen densities in the specimens subjected to 0.6-MeV and 0.95-MeV proton irradiation occurred at about 5 and 10 μm, respectively. Grazing incidence synchrotron reflection X-ray topography was performed using a monochromatic X-ray beam (λ = 0.15 nm) with a **g** vector of -1–128 or 11–28 at BL8S2 in the Aichi Synchrotron Radiation Center and BL20B in the Photon Factory at the High-Energy Accelerator Research Organization (details of the conditions are described in Ref.^47^). The X-ray topography observations enabled us to identify the Burgers vector of the dislocations^27,48,49^. The positions of BPDs propagating into the epitaxial layer were first determined, and then these positions were illuminated with UV light. A UV light emission diode with a wavelength of 365 nm was focused to a diameter of 3 mm, and the illumination intensity was adjusted to 10 W cm^−2^. During UV illumination, the specimens were heated to 373 K to expand the stacking fault by reference to the previous study^46^, and the temperature was measured by a radiation thermometer. The carrier lifetime of the specimens was evaluated by time-resolved photoluminescence (TR-PL) using a bandpass filter with a transmission wavelength of 370–410 nm, corresponding to luminescence from the band edge of 4H-SiC (~ 390 nm), as well as microwave photoconductivity decay (μ-PCD) with 10 GHz microwaves as a probe^12,50^. The excitation source for TR-PL and μ-PCD was a 266 nm pulsed laser with an injected photon density of 1 × 10^14^ cm^−2^.

## Results

**Figure 2:**
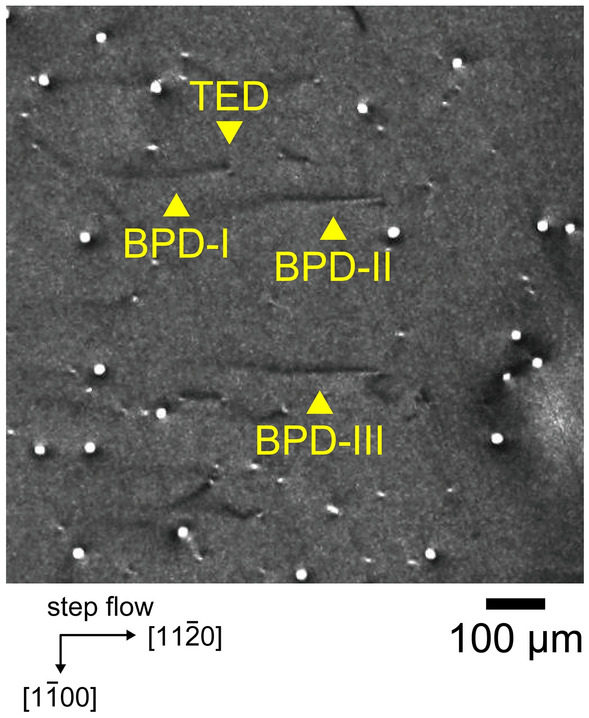
X-ray topography image of specimen without proton irradiation before UV illumination. BPD-I is converted to a TED, and BPD/TED conversion is not observed for BPD-II and BPD-III.

Figure 2 shows an X-ray topography image taken from a 4H-SiC epitaxial wafer without proton irradiation before UV illumination. Most of the linear features corresponding to BPDs were accompanied by small circular features corresponding to TEDs at the right end of the BPDs (e.g., BPD-I). On the other hand, some BPDs were not converted to TEDs and propagated into the epitaxial layers (e.g., BPD-II and BPD-III). The latter BPDs can be recognized by their characteristic contrast near the surface (on the right side in Fig. 2). The contrast of a BPD was reported to depend on the depth below the crystal surface in 4H-SiC, based on a comparison of ray-tracing simulation and X-ray topography results^51^. A BPD located less than about 5 μm from the surface (right side in Fig. 2) is imaged as a bright line bordered by dark lines, while a BPD located deeper (left side in Fig. 2) is imaged simply as a dark line. Therefore, it is possible to judge whether the BPD was converted or not. We carefully checked the positions of the propagated BPDs and illuminated their positions with UV light.

**Figure 3:**
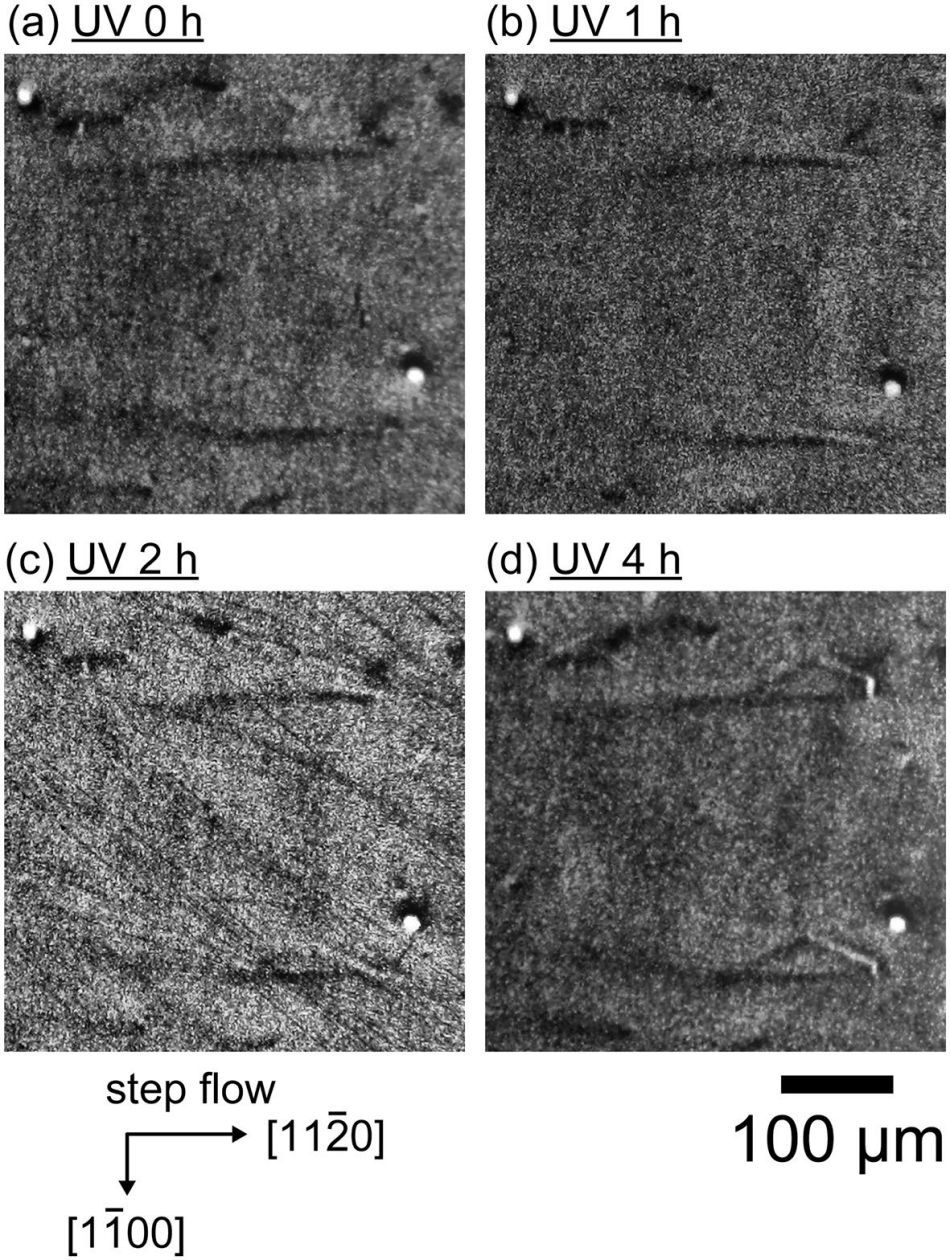
X-ray topography images of specimen without proton irradiation with different UV illumination times (a: 0 h, b: 1 h, c: 2 h, d: 4 h) with a power density of 10 W cm^−2^ at 373 K.

The evolution of 1SSF expansion in the epitaxial wafer during UV illumination with a power density of 10 W cm^−2^ without proton irradiation is shown in Fig. 3. In this X-ray topography geometry, SFs never exhibit contrast and the surrounding partial dislocations appear dark or bright depending on the direction^52^. Upon UV illumination, line contrast appeared and the surrounding area corresponding to the SF expanded as the UV illumination time increased. After further UV illumination with power densities of 5, 2.5 and 1 W cm^−2^ for 1, 2 and 12 h, we also observed the expansion of SFs. The current SF expansion behavior was consistent with that reported by Tanaka et al.^46^.

**Figure 4:**
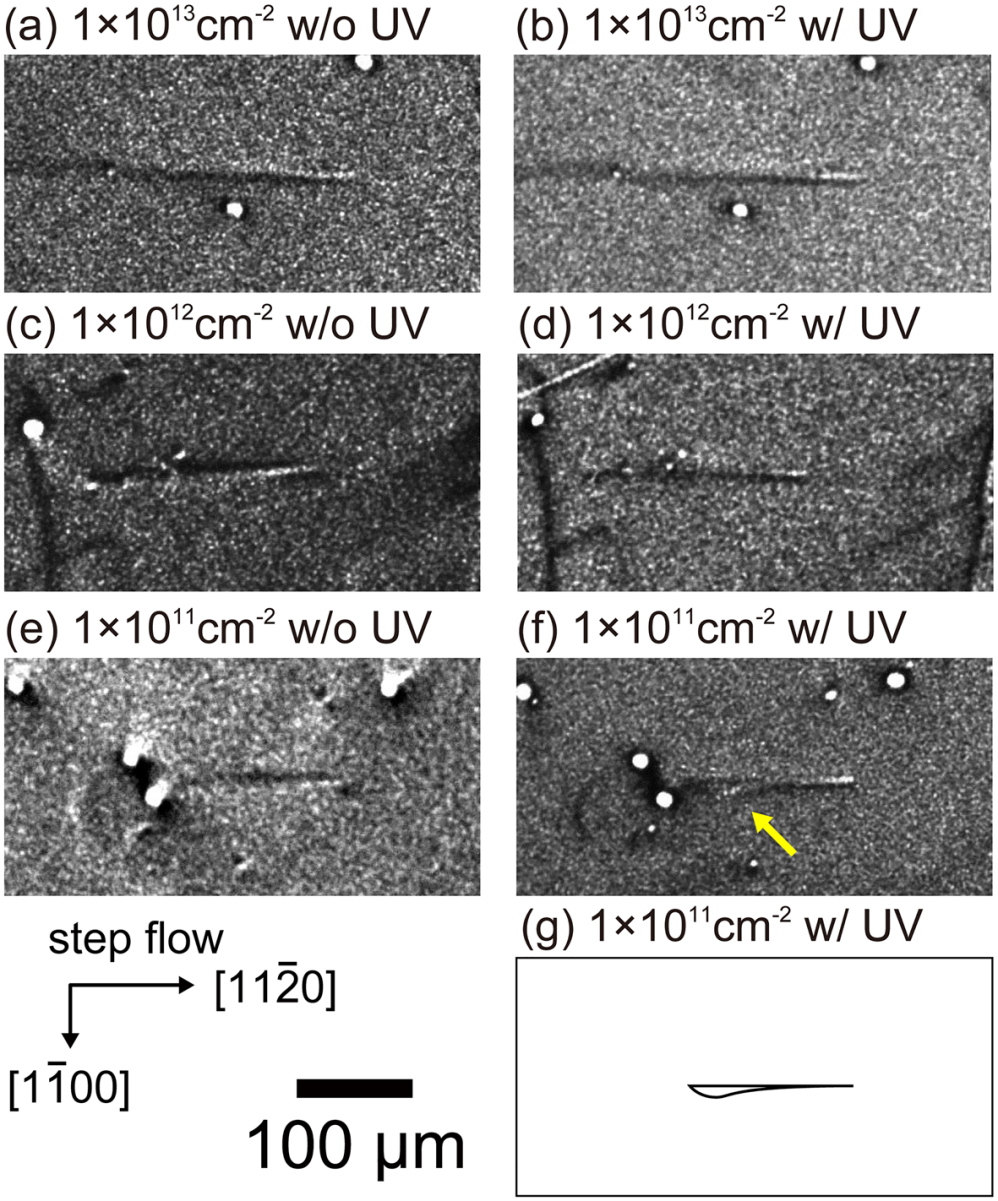
(**a**)–(**f**) X-ray topography images of specimens with proton irradiation fluence ranging from 1 × 10^11^ to 1 × 10^13^ cm^−2^ before and after UV illumination with power of 10 W cm^−2^ for 120 h and (g) schematic illustration of the shape of the SF shown in (**f**).

Figure 4 shows X-ray topography images taken from specimens with 0.6-MeV proton irradiation before and after UV illumination with a power density of 10 W cm^−2^ for 120 h. Even after UV illumination for 120 h, no apparent SF expansion was noticed except for a specimen that was subjected to proton irradiation at a fluence of 1 × 10^11^ cm^−2^, in which slight expansion of a SF was observed at the position indicated by the yellow arrow in Fig. 4f. Expansion of the SF only occurred at the left side of the BPD, indicating that it took place at the bottom side of the epitaxial layer and that proton irradiation suppressed SF expansion at the top side. These expansion behaviors were confirmed for 1 or 2 other BPDs in each specimen apart from those shown in Fig. 4.

**Figure 5:**
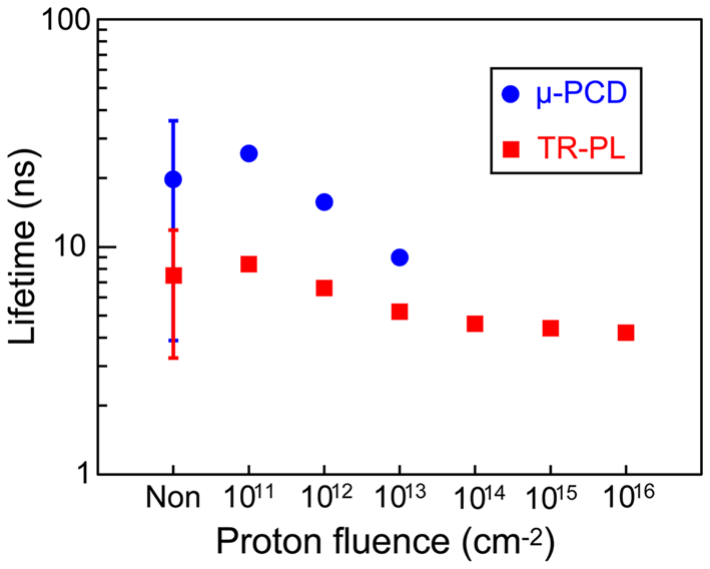
Carrier lifetime dependence on proton fluence measured by TR-PL and μ-PCD. The number of the measured specimens were 4 for non-irradiated specimens and 1 for other specimens.

The lifetimes measured by TR-PL and μ-PCD for specimens subjected to 0.95-MeV proton irradiation with different fluences are shown in Fig. 5. We conducted lifetime measurements on 4 different specimens for a 4H-SiC epitaxial wafer without proton irradiation. Although the absolute lifetime values differ depending on the measurement method (the lifetime measured by μ-PCD was always larger than that by TR-PL), the same tendency, in which the lifetime is almost unchanged by the proton fluence of 1 × 10^11^ cm^−2^ and decreases with increasing proton fluence, was observed for both TR-PL and μ-PCD measurements. Note that the lifetime had a distribution in the epitaxial wafer, resulting in large deviations in the lifetime values for the non-irradiated specimens; also, the lifetime tended to be small near the edge of the wafer, where propagation of BPDs was frequently observed.

### Driving force of SF expansion by UV illumination

The excess carrier concentration in epitaxial layers ($\Delta n$) caused by UV illumination in the steady state was estimated by the following equation for low excess carrier concentrations, assuming that surface and interface recombination is negligible^53^:

1 $$-\frac{\Delta n}{{\tau }_{epi}}+G=0$$

where ${\tau }_{epi}$ is the bulk carrier lifetime in the epitaxial layer and *G* is the rate of generation of excess carriers in the epitaxial layer calculated from the photon flux and absorption^54^. The value of ${\tau }_{epi}$ is not the same as the carrier lifetime measured by μ-PCD and TR-PL^53^. Here, the typical value for an as-grown n-type SiC epitaxial layer (0.1–1 μs) was adopted for the estimation. Note that the measured lifetime was much smaller than typical value of the bulk carrier lifetime since the surface recombination largely influences on the measured carrier lifetime of the epitaxial layer with the thickness of 10 μm^53,55,56^. The excess carrier concentration was estimated to be 2 × 10^14^–2 × 10^15^ cm^−3^, which led to an electronic energy gain (Δ) of around 0.5 ~ 3 mJ/m^2^ from the result of calculations reported in Ref.^17^.

On the other hand, it was possible to estimate the driving force for SF expansion (negative stacking fault energy γ) caused by UV illumination from the radius of curvature (*R*) of pinned partial dislocations under an equilibrium of forces acting on the dislocation by the following equation^24^:

2 $$\gamma =-\frac{{b}^{2}}{4\pi R}\left\{{K}_{edge}{\mathrm{sin}}^{2}\theta +{K}_{screw}{\mathrm{cos}}^{2}\theta +2\left({K}_{edge}-{K}_{screw}\right)\mathrm{cos}2\theta \right\}\mathrm{ln}\left(\frac{{r}_{1}}{{r}_{0}}\right)$$

where *b*, *K*_edge_, *K*_screw_, *θ*, *r*_0_ and *r*_1_ are the Burgers vector of the dislocation, the energy factors for edge and screw components, the angle between the line vector and the Burgers vector, and the inner and outer cut-off radius, respectively. Judging from the shapes of the vertexes in Fig. 3d, the value of *R* ranged from 5 to 50 μm, corresponding to values of γ ranging from -0.6 to -6 mJ/m^2^. The SF energy without UV illumination (γ_0_) was estimated by the following relationship:

3 $$\gamma ={\gamma }_{0}-\Delta $$

From the obtained values of Δ and γ, the value of γ_0_ was evaluated as + 2 to -5 mJ/m^2^. This indicates that the SF energy without UV illumination near room temperature was much lower than the assumed value of γ_0_ (5 ~ 20 mJ/m^2^) based on the SF energy evaluated by the dissociation width of partial dislocations deformed at high temperature^17,25^, and probably be negative value, although the current estimation is a bit rough.

The shear stress acting on the partial dislocation as a result of the negative stacking fault energy was estimated to be 3–30 MPa, which is much lower than the bulk CRSS of 4.5 GPa for basal slip in 6H-SiC estimated by a micropillar compression test^37^. This indicates the occurrence of a drastic decrease in activation energy due to the recombination-enhanced dislocation glide induced by UV illumination, as was reported for carrier injection and electron beam irradiation^13,35^.

### Effect of proton irradiation

TR-PL and μ-PCD measurements indicated that the carrier lifetime was decreased by proton irradiation, as was reported by Hazdra et al.^44^ They reported that carrier lifetime reduction took place due to the introduction of *Z*_1/2_ centers, which are related to carbon vacancies. In the present study, 0.6-MeV proton irradiation at fluences ranging from 1 × 10^12^ to 1 × 10^13^ cm^−2^ resulted in an evident reduction in carrier lifetime and suppression of SF expansion, which was in good agreement with the results reported by Tawara et al., who demonstrated that a short carrier lifetime can successfully suppress SF expansion^34^. On the other hand, 0.6-MeV proton irradiation at a fluence of 1 × 10^11^ cm^−2^ also resulted in suppression of SF expansion even though the carrier lifetime was almost unchanged. This implies that proton irradiation affects not only the carrier lifetime but also dislocation motion. The interaction between dislocations and point defects, such as protons and vacancies, would increase the CRSS for the glide of partial dislocations. Kwon et al. reported that an increase in the CRSS occurred with proton irradiation based on micro-pillar compression testing of 6H-SiC crystals^57^.

**Figure 6:**
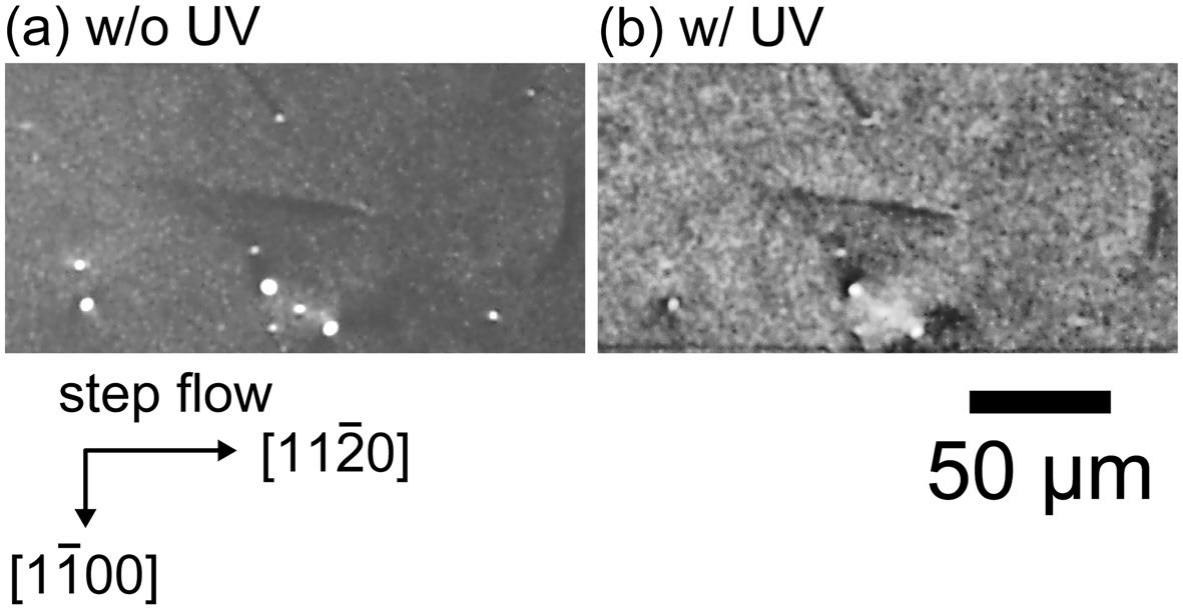
X-ray topography images of epitaxial layer with thickness of 5 µm, nitrogen concentration of 6 × 10^15^ cm^−3^, and 0.3-MeV proton irradiation at a fluence of 1 × 10^15^ cm^−2^ after annealing at 1973 K for 1 h. X-ray topography images were taken (**a**) before and (**b**) after UV illumination on the specimen with power of 10 W cm^−2^ at 373 K for 10 h.

To further confirm the effect of proton irradiation on dislocation glide, we investigated SF expansion for a specimen subjected to proton irradiation after high-temperature annealing to recover the carrier lifetime. Figure 6 shows an X-ray topography image taken from an epitaxial layer with a thickness of 5 µm, a nitrogen concentration of 6 × 10^15^ cm^−3^, and subjected to 0.3-MeV proton irradiation at a fluence of 1 × 10^15^ cm^−2^ after annealing at 1973 K for 1 h. For this specimen, μ-PCD measurements showed that the carrier lifetime was unchanged after annealing. The propagating BPD in the epitaxial layer was not expanded to a SF even after UV illumination with a power density of 10 W cm^−2^ for 10 h at 373 K, as shown in Fig. 6. These results indicate that proton irradiation increases the CRSS for the glide of partial dislocations under UV illumination and suppresses SF expansion. Hydrogen (proton) passivation of surfaces, point defects and dislocations in semiconductor crystals, including silicon and 4H-SiC, have been reported by many researchers^58–61^. If protons terminate the dangling bonds at the core of partial dislocations in an epitaxial layer, the mobility of partial dislocations is expected to decrease and recombination at dislocations is expected to be suppressed, resulting in suppression of recombination-enhanced dislocation glide under UV illumination. The current results imply that bipolar degradation in SiC power devices would be suppressed by proton irradiation, which has good compatibility with semiconductor processing.

## Conclusion

SF expansion in n-type 4H-SiC epitaxial layers subjected to UV illumination and different levels of proton irradiation was investigated by X-ray topography as well as carrier lifetime measurements. The results obtained are summarized as follows:

1. Under UV irradiation, SFs expanded from BPDs in a 4H-SiC epitaxial layer without proton irradiation, but the expansion was suppressed by proton irradiation at fluences ranging from 1 × 10^11^ to 1 × 10^13^ cm^−2^.
2. From the radius of curvature estimated from X-ray topography observations, the SF energy without UV illumination at 373 K was evaluated as + 2 to − 5 mJ/m^2^, which was much smaller than the expected value (5 to 20 mJ/m^2^) based on the SF energy evaluated from the width of partial dislocations deformed at high temperature
3. The excess carrier lifetime measured by both TR-PL and μ-PCD was reduced by proton irradiation at fluences larger than 1 × 10^12^ cm^−2^.
4. SF expansion in 4H-SiC epitaxial layers with proton irradiation at a fluence of 1 × 10^11^ cm^−2^ was suppressed without any evident reduction in the excess carrier lifetime, which implies that recombination-enhanced dislocation glide is hindered by interactions between partial dislocations and point defects introduced by proton irradiation.
5. SF expansion was also suppressed even after high-temperature annealing to recover the excess carrier lifetime, which supports the hypothesis that proton irradiation hinders recombination-enhanced dislocation motion.

The present results imply that the problem of bipolar degradation in SiC power devices could be solved by proton irradiation, which has good compatibility with semiconductor processing.

## Data Availability

All data generated or analyzed during this study are included in this published article.
